# “Asking for help, quite a challenge”. Time from onset of symptoms to consultation with a psychiatrist

**DOI:** 10.1192/j.eurpsy.2023.1005

**Published:** 2023-07-19

**Authors:** T. Jiménez Aparicio, M. Fernández Lozano, M. Merizalde Torres, E. Domínguez Guerra, A. Rodríguez Campos

**Affiliations:** 1 Servicio de Psiquiatría; 2 MFyC, Sacyl, Hospital Clínico Universitario Valladolid, Valladolid, Spain

## Abstract

**Introduction:**

One of the biggest challenges for primary care professionals is to know when it is appropriate to request a consultation with a psychiatrist. A complete medical history should be performed to detect anxious-depressive symptoms, as well as to determine the intensity, the trigger, time of evolution, and the functional repercussion (1). It is also important that the patient is able to express his or her symptoms and ask for help. The concept of “Alexitimia” refers to the difficulty of expressing feelings verbally, and is a frequent symptom in depressive patients (2).

In mild cases and with little repercussion, the physician himself can initiate treatment and follow up (3). However, on other occasions, it will be advisable to request a consultation with psychiatry.

**Objectives:**

The main objective is to observe the time that elapses from the onset of symptoms until consultation with the Mental Health team is finally requested. Some preliminary results can already be obtained from this data collection.

**Methods:**

We have decided to carry out a descriptive study, collecting different variables from patients attending a first Psychiatry consultation.

**Results:**

In a total sample of 208 patients, the majority (67%) were between 31 and 60 years old. Following the DSM-V criteria (4), patients were classified into groups according to their disorder: Adaptive, depressive, or other. These data were cross-referenced (Figure 1).

Subsequently, the time elapsed from the onset of symptoms (referred by the patients) was collected, until the referral to Psychiatry was processed. In order to make a comparison, average time (in days) was calculated for the different groups according to their age and diagnosis.

Those patients under 30 years were referred to psychiatry later. A downward trend was seen as the age range increased. In the “younger than 30” and “between 31 and 60” groups, patients who met criteria for Depressive Disorder took longer to be referred, which was striking considering that they are usually considered as more severe patients (Figure 2). This can be attributed to a greater difficulty in expressing their emotions (alexithymia), as another depressive symptom. Disaggregating these data by gender, the patients who clearly took the longest to be referred were men under 30 years old with a final diagnosis of Depressive Disorder (Figure 3). This gender difference is consistent with the social impact of alexithymia according to some articles (5).

**Image:**

**Figure:**
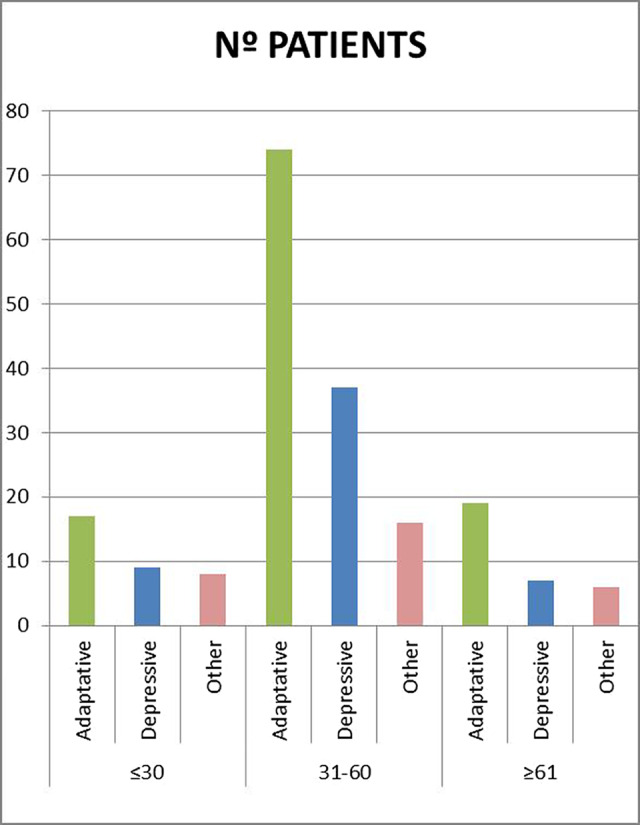

**Image 2:**

**Figure:**
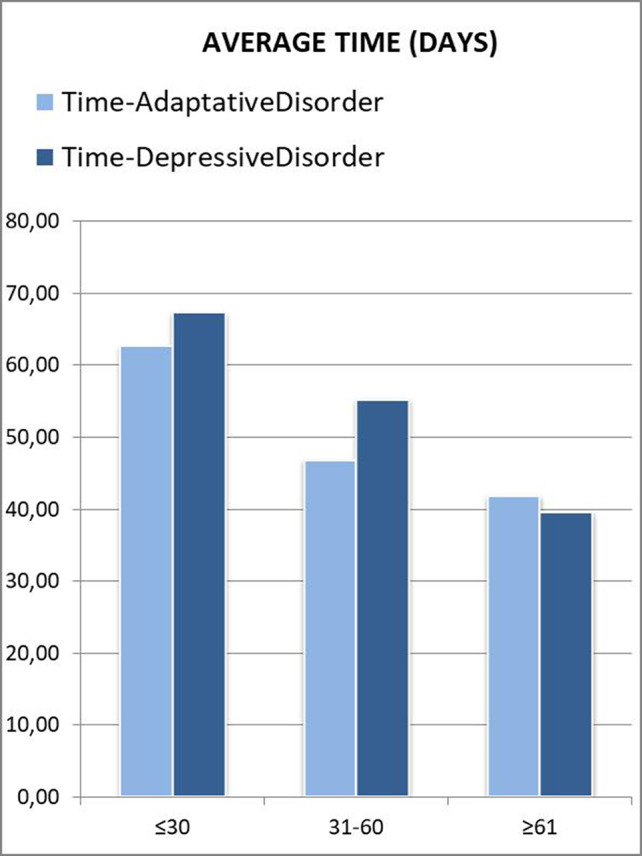

**Image 3:**

**Figure:**
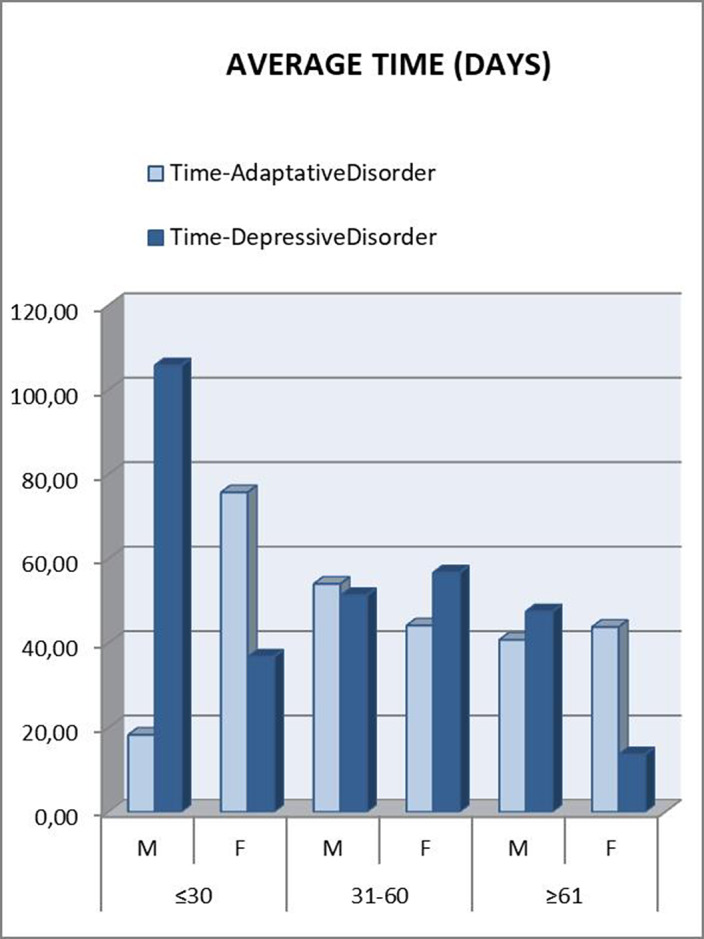

**Conclusions:**

It is important that primary care physicians know how to take a complete history in those patients with symptoms of anxiety and depression.

In many cases, patients themselves have difficulties expressing their emotions and feelings (alexithymia), which may be another symptom of their discomfort.

This may lead to a delay in the time until referral to a psychiatry is requested, and therefore a worsening of symptoms.

**Disclosure of Interest:**

None Declared

